# Biomimicry Industry and Patent Trends

**DOI:** 10.3390/biomimetics8030288

**Published:** 2023-07-03

**Authors:** Haejin Bae

**Affiliations:** Ecological Technology Research Team, Division of Ecological Application Research, National Institute of Ecology, Seocheon-gun 33657, Republic of Korea; hjbae@nie.re.kr

**Keywords:** patent technology, biomimicry trend, biomimetic technology, biological characteristics

## Abstract

This study examines the current technological level and industrial/technical trends in the field of biomimicry technology, as well as recent technological and research and development trends. Patent analysis was conducted, focusing on technology that uses design elements and biological/ecological characteristics to provide solutions to technological problems. The technological scope of the analysis included the field of technologies and materials that apply to the conditions found in ecology, as well as robot machines and devices designed to mimic certain animals and ecological elements. The search for patents was conducted in Korea, the United States, Japan, and Europe from 1975 to 2021, resulting in a total of 8278 raw data cases, from which 940 valid patents were selected. The percentage of patent document and the status of both domestic and foreign applicants varied among the countries of Korea, the United States, Japan, and Europe. Based on the results of the patent analysis, it was found that biomimicry technology is in a growth phase that is expected to continue in the future and that Korea and the United States are leading the development of this technology.

## 1. Introduction

Biomimicry refers to studying nature, ecology, and living system to find solutions to problems faced by humanity, and it could provide sustainable, efficient, and innovative solutions [1]. Biomimicry has the potential to transform the majority of domestic and international economies, making it possible to achieve both economic growth and environmental goals simultaneously [2,3]. Although the commercial application of biomimicry is still irregular, several well-known achievements, such as Shinkansen and Velcro, demonstrate the potential of biomimicry [4,5,6]. The public’s awareness of patents related to bio-inspired and biomimetics likely arose when it was revealed that the roof design of the Crystal Palace in London, United Kingdom, was bio-inspired by the functionality of the leaf of a water lily, specifically its ability to support substantial weight in water [7,8]. A catalyst for raising public awareness of patents related to ecological imitation was George Mestral’s invention of VELCRO^®^ in 1955 [9]. The patent analysis related to biomimicry, biomimetics, and bio-inspired were shown in a few studies [10,11,12,13,14,15], but they were still not sufficiently provided for the patent landscape to show technological trends. Moreover, it was challenging to find the latest patent analysis in the fields. Interestingly, there were research papers that specifically focused on methods for patent search and data extraction [16,17,18,19,20].

Over the past 20 years, global interest in biomimicry has increased, while public awareness of this field remains low [21]. To advance biomimicry, patent data based on numbers are needed to change the perception of the public and investors towards biomimicry-related fields. In particular, biomimicry has the potential to connect South Korea’s economic growth goals with environmental protection. Therefore, new designs, processes, materials, products, and systems through biomimicry could greatly transform various industries [22,23,24,25]. As a result, it is possible to optimize product production through improved designs, the use of environmentally friendly materials, and minimizing costs and energy while also creating new markets [26,27,28,29]. Furthermore, the concept of drawing inspiration from nature in biomimicry could help companies fulfill broader social responsibilities demanded by ESG (environmental, social, governance) indicators worldwide while also achieving financial goals [30,31]. The world could lead these changes and reap the benefits.

The development of biomimicry by providing key findings and information on biomimicry through statistical data on biomimicry with a focus on Korea was promoted. The current technology level and industrial/technical trends in the field of biomimicry technology for the continuation of the biomimicry patent trend analysis was identified, and recent trends in technology research and development are analyzed. Many countries, especially Korea, Japan, and USA, often encounter issues related to technology convergence or new technology trends. In the case of Korea, there has been a consistent improvement in both quantitative and qualitative aspects of patent applications in line with the industrial technology trends based on the reports from the Korea Institute of Intellectual Property [32]. 

This study seeks to discover R&D trends by country and technologies of major applicants by analyzing patent trends. Furthermore, this report gives objective information on future research and development trends and research directions of biomimicry technology through a quantitative analysis to conduct an overall analysis of trends, which is divided into the patent landscape for technology and a key applicant analysis.

Comparative and analytical papers on patents related to biomimicry technologies and industries are not easily found. Therefore, this study provides unique and valuable information to better understand the overall trends in biomimicry fields.

## 2. Materials and Methods 

### 2.1. Scope of Analysis

For patent analysis, it was searched for patents filed, disclosed, and registered in South Korea, the United States, Japan, and Europe from January 1975, when patent documents began to be documented, until December 2021. In terms of sheer numbers, Chinese patents are incredibly diverse and abundant, which could lead to distortions when conducting comparative analyses with other countries. It was not excluded from the analysis just because the number of Chinese patents was enormous, but when the trends in Chinese patents and patents of other countries were listed in the raw data analysis, the meaning of comparison was not apparent. Therefore, Chinese patents were excluded from the analysis in this study. It was performed for a patent search using the WINTELIPS search DB, an online patent search service developed by WIPS Co., Ltd. (Seoul, Republic of Korea) [33]. Patents searched in the patent database included not only registered patents for which the patent document was still valid but also patents in the publication, rejection, withdrawal, and examination states. Data were classified for patent documents. The analysis is based on the application date. In order to obtain reliable data for patents applied from June 2020, when undisclosed data still exists, quantitative meaning is not valid when analyzing patents. Thus, only the data until 2019 was viewed as valid data that has quantitative meaning. The analysis was conducted based on the filing date, and generally, it is considered as patents that had been publicly disclosed without any legal issues approximately 18 months after the filing of the patent document as the basis for our analysis.

### 2.2. Technical Scope of Target Technologies and Valid Patent for Analysis

Patent analysis was undertaken with a focus on technology that presents solutions to technological challenges by borrowing design features found in nature or characteristics of living organisms. The core keywords were derived from the content of biomimicry technology, and the search formula was built and enlarged four times by merging the derived keywords. The number of extended patent families related to the derivation of keywords and the generated search query was a total of 8278 combinations of keywords over 4 iterations to extract the data (Supplementary Materials Table S1), and representative patents were provided (Supplementary Materials Table S2). The numbers of raw data produced by applying the final search formula on the search DB were 1884 for Korea, 908 for Japan, 4994 for the USA, and 542 for Europe. Noise reduction criteria were established from the raw data collected by using the keywords and search formula derived above to eliminate from the analysis patents irrelevant to the target technology that is the topic of this report. If the following main content was present, it was considered noise and excluded from valid patents: artificial lures/mimics resembling living organisms, eco-compatible materials/compounds, pharmaceutical compositions, and the like.

Valid patents were derived from these criteria, and quantitative analysis was performed for the derived valid data. The numbers of valid patents were 287 for Korea, 88 for Japan, 472 for the USA, and 93 for Europe. 

The quantitative analysis is separated into two parts: the patent technology landscape and the analysis of major applicants. Regarding the patent technology landscape, the stages of patent technology growth were analyzed through the current status of technology development activities, the trend in domestic and foreign patent documents, the number of applications filed by section, and the degree of increase or decrease in the number of applications in major countries such as South Korea (KIPO), USA (USPTO), Japan (JPO), and Europe (EPO). For the analysis of major applicants, multiple top applicants were derived. The applicants’ capacities to secure technology, their primary technical fields, and the density of patent documents were analyzed.

## 3. Results and Discussions

### 3.1. Patent Document Trends in Major Countries by Year

The analysis was to identify leading countries in the target technology field based on the share of patent technology applications by country in South Korea, the United States, Japan, and Europe. Compare quantitative trends in patent technology applications by country from the past to the present to identify the technological position of South Korea in comparison to other countries.

**Figure 1:**
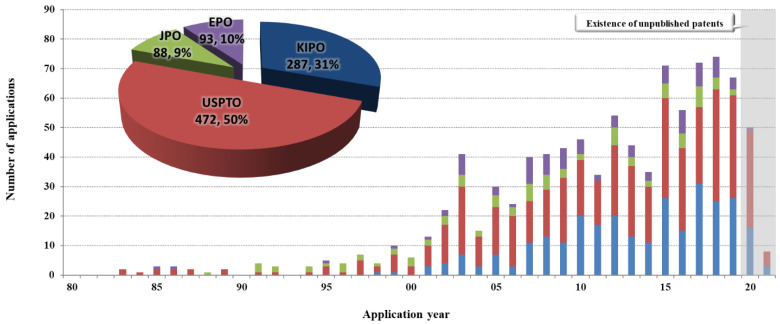

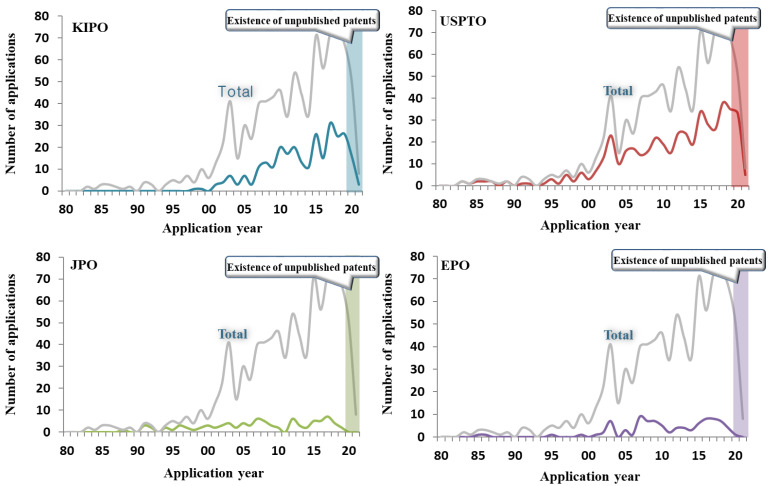
Patent trends in major applicant countries by year.

The total patent trend in the target technologies has shown fast growth since 2000 (Figure 1). A rapid growth in patent documents relating to the target technologies appears in the patent trend. Thus, it seems that the rapid increase in applications in South Korea and the United States since the mid-2000s is leading the general trend. In Japan and Europe, however, the number of applications is repeating growing and dropping cycles, but this does not appear to have a substantial effect on the overall trend.

In terms of the status of patent ownership by each country for the target technologies, 472 patents were applied in the United States (USPTO) (50%), 287 in South Korea (KIPO) (31%), 88 (9%) in Japan (JPO), and 93 in Europe (EPO) (10%). Thus, the United States has applied for the largest number of patents regarding this technology. There were almost no applications from the beginning of the analysis period until 2000 in South Korea and the United States, but the number of applications surged after 2000, resulting in them leading the trend until 2019. South Korea (KIPO) appears to have applied for 287 patents in the target technologies. There were no patent documents from the beginning of the analysis period until 2000, but there has been steady growth from 2001 until 2019.

From 2001, when applications began to increase, until recently, South Korea’s patent trend was similar to the general patent trend, and it appears to dominate the technology market in the recent section alongside the United States. Because the quantitative flow of South Korean patent technology differs from that of Japan and Europe, it is difficult to determine whether the technology level is comparable. It is possible to find out later by discovering the details of the patented technology through in-depth analysis. The patent trend in South Korea indicates steady growth without a dramatic spike or fall. However, there were temporary declines in 2004, 2006, 2013, and 2017.

The United States (USPTO) applied for 472 patents in the target technologies. The trend of gradually increasing patent documents has been maintained from the beginning of the analysis period to the present. The United States appears to be influencing the patent document increase and decrease trend since 1995. At the start of the analysis period, the United States had a comparable number of applications as Japan. However, unlike Japan, where applications have been continuously decreasing since 2003, applications in the United States have been steadily growing until recently.

The number of applications dropped dramatically temporarily in 2010. It is thought that there was a temporary drop in applications in 2009 due to a reduction in government spending. In target technologies, Japan (JPO) has filed 88 patents. Applications have been filed intermittently since 1995, but the number of applications increased slightly from the 2000s. However, it appears that around five applications are continuing while the increase and decrease cycles are repeated. The trend in applications was similar to that of the United States until 2000, but the number of applications has remained around five cases without growing since 2000. There have been significantly decreasing sections since 2006. This is considered to be owing to a change in Japan’s Intellectual Property Office’s patent document policy in 2006, which urged action from the industry to carefully choose quality above quantity when filing for patents.

There have been 93 patents in Europe (EPO) in target technologies. The number of applications has undergone repeated rises and declines during the analysis period. European patents are not directly applied for by individual European countries but rather by designating specific countries through the European Patent Office (EPO). As a result, they have more technical value, a higher possibility of product connection, and a larger family size than patents in other nations. Furthermore, it can be asserted that global policy has a significant impact. In Europe, the number of applications surged after 2000, followed by a trend of repeated increases and decreases. The size of the family per application is characterized by significant differences between large increases and decreases. No section leading the rise and decline trend in the entire patent technologies exists. However, given the technical significance of European patents, it is worthwhile to investigate the application trend in multi-application organizations and global companies that have entered Europe.

### 3.2. Application Trends of Locals and Foreigners in Major Countries

**Figure 2:**
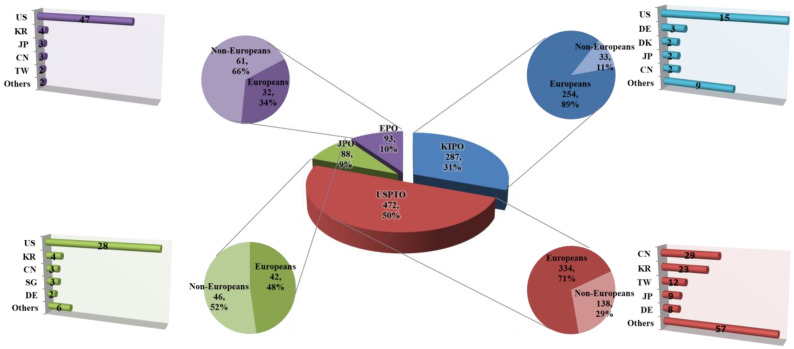
Patent document status of locals and foreigners in major applicant countries (1980–2021).

**Figure 3:**
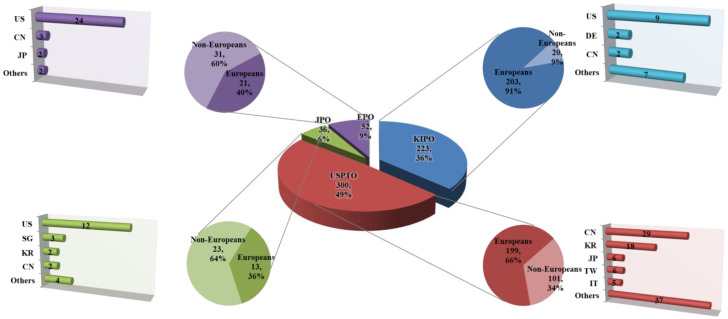
Patent document status of locals and foreigners in major applicant countries over the last decade (2011~2021).

The distribution of applications filed by locals and foreigners by classifying the nationalities of applicants by country in South Korea (KIPO), the United States (USPTO), Japan (JPO), and Europe (EPO) were identified. The number of applications filed by foreigners by nationality by country to infer the inflow of foreign technology into the country, dependence on foreign technology, and the technological power of the country was analyzed. The number of locals and foreigners’ applications by country from the past to the present to identify changes in the number of locals and foreigners leading technology development within the country were compared. The number of patents filed in target technologies by country was 287 cases for South Korea (KIPO), 472 cases for the USA (USPTO), 88 cases for Japan (JPO), and 93 cases for Europe (EPO) (Figure 2). The detailed status of patent applications by domestic and foreigners is shown in Supplementary Materials Table S3. The current status of domestic and foreign patent applications for the past 10 years was representative of 223 cases for South Korea (KIPO), 300 cases for the USA (USPTO), 36 cases for Japan (JPO), and 52 cases for Europe (EPO) (Figure 3). The analysis concluded that the proportion of domestic and foreign patent documents by country gauges the globalization of their respective technology markets. When assessing the attractiveness of the technology market, Korea appeared to have a lower level compared to the United States and Japan, while Europe showed a high level of the market. In addition, as a result of comparing the patent competitiveness index and technological influence by applicant’s nationality, the patent impact index was high in France, Canada, and USA, while the technology strength was high in USA, Korea, and Japan, in order. Furthermore, when the applications for registered patents were analyzed, SpinCore Technology INC, as a US national applicant, ranked first, followed by universities in USA and Korea (data were not shown). The trend could be seen as consistent with the results from the US patent related to biomimetics and bionics had increased from 1985 to 2005 [16]. This analysis showed the patent trends in biomimicry technology by combining various keywords. However, upon closer examination, patents explicitly named as biomimicry technology or similar terms are rare in the titles of patents. In the future, focusing on patents that specifically involve biomimicry technology and commercial examples would provide more useful information. 

### 3.3. Patent Document Status of Locals and Foreigners

**Figure 4:**
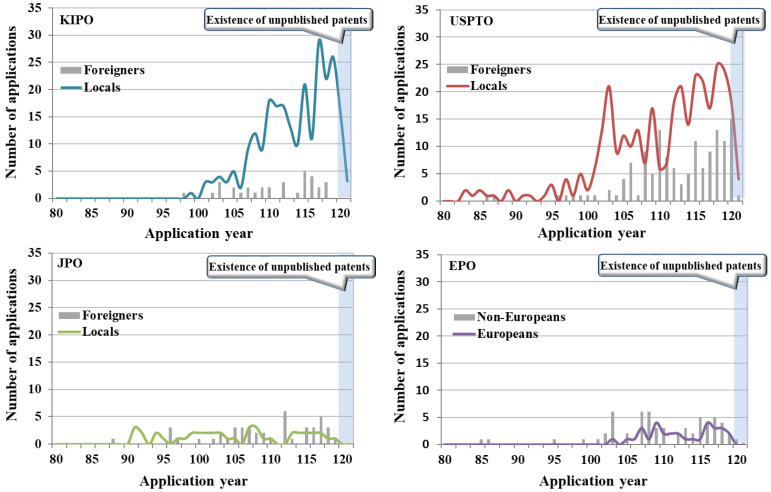
Patent document status of locals and foreigners in major applicant countries by year.

The patent documents by locals and foreigners in South Korea (KIPO) were 89% (254 cases) for locals and 11% (33 cases) for foreigners (Figure 4). In comparison with South Korea’s typical patent document tendency with a local application rate of about 70–75% in other technology fields, the target technologies showed a strong tendency of applications by locals. This is highly similar to the ratio of applications by locals in the last decade. In the current status of locals and foreigners’ applications for patent technologies in South Korea (KIPO), the high proportion of applications by locals has been maintained without an inflection point in the graph of the proportion of locals and foreigners’ applications from the beginning of the analysis period to the present. In particular, the gap in the proportions of locals and foreigners tends to widen gradually in the recent section. This suggests strong local-centered R&D characteristics of the target technologies. Regarding the nationalities of foreigners who applied in South Korea (KIPO), applicants with USA nationality applied for the largest number of patents in all sections. 

In addition, German, Danish, Japanese, and Chinese applicants applied for patents in South Korea. The trend in the recent decade was similar to the overall trend. The ratio of locals and foreigners filing patent documents in the United States (USPTO) was 71% (334 cases) for locals and 29% (138 cases) for foreigners. When compared to the US patent document trend with a local application rate of around 65%, which commonly appears in other technical fields, the United States also showed an application trend centered on locals, as in South Korea. However, the proportion of local applicants in the last decade was 66%, suggesting that the proportion of foreigners’ applications has increased compared to the past.

In the patent technology application status by locals and foreigners of the United States (USPTO), the proportion of applications by locals was higher than the proportion of applications by foreigners from the beginning of the analysis period. From 1995 to 2010, the proportion of applications by locals tended to outnumber that of foreigners. Since 2010, the number of applications by foreigners has increased slightly, narrowing the gap between locals and foreigners. The United States (USPTO) also showed characteristics of research and development centered on locals compared to other technology fields, but the ratio of research and development by foreigners was higher than that of South Korea (KIPO). The United States also did not show the point of inflection in the graph of the proportion of applications by locals and foreigners. Regarding the nationalities of foreigners who have applied for patents in the United States (USPTO), Chinese applicants have applied for the largest number of patents. In addition, applicants from South Korea, Taiwan, Japan, and Germany applied for patents in the United States. In the last decade, new countries such as Italy have entered the market. 

The proportion of locals and foreigners filing patent documents in Japan (JPO) was 48% (42 cases) for locals and 46% (52 cases) for foreigners. In comparison with Japan’s patent document tendency with a local application rate of around 85%, which is common in other technology fields, the target technologies showed a strong tendency for applications by foreigners. The proportion of applications by locals in the last decade was 36%, suggesting that the proportion of applications by locals is further decreasing compared to the past. In the patent technology application status of locals and foreigners in Japan (JPO), the inflection points of the graph line for the proportion of locals and foreigners tended to fluctuate a lot throughout the analysis period. The proportions of applications by locals and foreigners showed an equal trend. The nationalities of foreigners who applied for Japan (JPO) showed that applicants of United States nationality applied for the largest number of patents. In addition, applicants from South Korea, China, Singapore, and Germany applied for patents in Japan. A trend similar to this has been observed in the last decade.

The proportion of patent documents by locals and foreigners in Europe (EPO) was 34% (32 cases) for locals and 66% (61 cases) for foreigners. In European patents, locals refer to applicants of EU member nationality, and foreigners refer to applicants of other nationalities.

For European patents in target technologies, the proportion of foreigners was about twice as high as that of locals. The rate of applications by locals in the last decade was 40%; thus, the proportion of applications by locals has increased compared to the past. In the current status of applications by locals and foreigners for patent technologies in Europe (EPO), the inflection point of the graph of the proportions of applications by locals and foreigners repeatedly appears from the beginning of the analysis period to the latest. This suggests that the dependence of patent technologies on locals and foreigners is constantly changing due to the nature of European patents. Regarding the nationalities of foreigners who applied for Europe (EPO), applicants of US nationality applied for the majority of patents. Applicants from South Korea, Japan, China, and Taiwan also applied for European patents. A similar trend was observed in the last decade. 

### 3.4. Patent Technology Growth Stage

**Figure 5:**
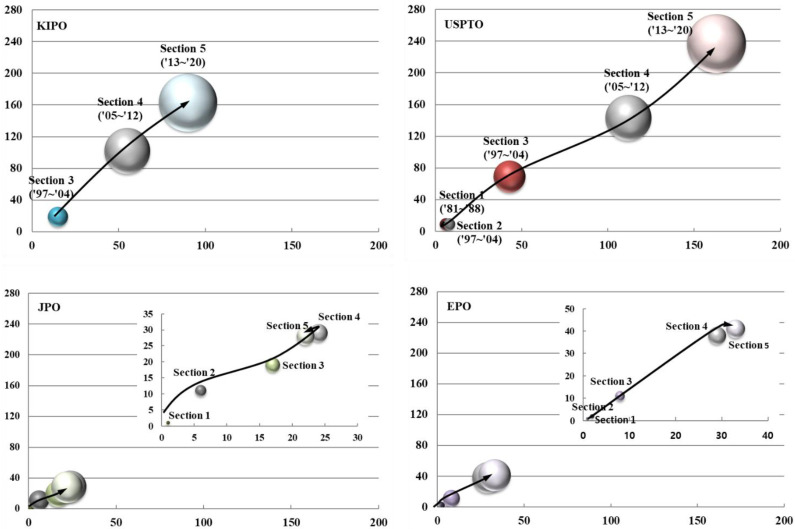
Patent technology growth stages of major countries.

To analyze the patent technology growth stages of target technologies, the entire analysis period of 40 years was divided into five periods of 8 years. In Figure 5, Section 1 (1981–1988), Section 2 (1989–1996), Section 3 (1997–2004), Section 4 (2005–2012), and Section 5 (2013–2020). The growth stage of target technologies based on patent technologies can be interpreted as a period of growth in which both the number of applications and applicants increase on the graph. In particular, the number of applications and applicants tends to increase significantly from Section 3 to Section 4. Overall, it exhibits the shape of the growth stage.

The patent technology growth stage of South Korea (KIPO) can be interpreted as a period of growth in which the number of applications and applicants increased. The number of applications and applicants showed a significant increase from Section 3 to Section 5. However, there was no application from Section 1 to Section 3. The target data for South Korea’s patent technology growth stage analysis consists of 284 cases; thus, the validity of the analysis can be acknowledged.

The patent technology growth stage of the United States (USPTO) can be interpreted as a period of growth in which the number of applications and applicants increased. The number of applications and applicants is on an increasing trend from Section 1 to the latest section. The US patent technology growth stage appears similar to the overall growth stage. In particular, the shape of the overall growth stage from Section 3 to Section 4 is highly similar to the shape in which significant technology growth occurs. The data subject to analysis of the US patent technology growth stage consists of 467 cases. Thus, the validity of the analysis can be acknowledged.

The patent technology growth stage of Japan (JPO) can be interpreted as a period of maturity in which both the numbers of applications and applicants decreased after showing a growth stage until Section 4. The number of applications decreased slightly from Section 4 to the latest section, indicating that it has entered the maturity stage. Japan’s patent technology growth stage shows a similar pattern to the overall growth stage from Section 1 to Section 4, but it shows a pattern different from the overall growth stage after Section 4. The target data for analysis of Japan’s patent technology growth stage consists of 88 cases. Thus, it is rather unreasonable to say that the validity of the analysis can be acknowledged.

The patent technology growth stage of Europe (EPO) can be interpreted as a period of growth in which both the number of applications and applicants increased. The number of applications and applicants increased greatly from Section 1 to Section 4, and the number of applications and applicants increased slightly from Section 4 to Section 5. The shape of Europe’s patent technology growth stage is similar to the overall growth stage from Section 1 to Section 4. However, there is a short break between Section 4 and Section 5. The target data for analysis of Europe’s patent technology growth stage consists of 93 cases. Thus, it is rather unreasonable to say that the validity of the analysis can be acknowledged. In the growth stage of target technologies, the increase in the number of applications and applicants continues for an extended period from Section 1 to the latest section. In the later sections, there is room for maintaining the same shape or entering the maturity stage in which the number of applications decreases while the number of applicants is slightly increased or maintained.

The patent technology growth stage of South Korea (KIPO) appears to be most similar to that of the United States (USPTO). The graph shows that the growth rate of South Korea’s technology is almost equal to that of the United States.

In this analysis, most technology and industrial sectors are experiencing growth, leading to an increase in the influx of various new applicants and subsequent application activities. It is determined that the barriers to entry in the technology sector are low, and there is a high potential for success among new entrants. 

### 3.5. Patent Share and Growth Rate of Major Countries

This study found the patent document trend, technology characteristics, and relative prospect for each detailed technology by simultaneously considering the patent share and the growth rate of each detailed technology. The patent share and the growth rate of detailed technologies by country among Korea (KIPO), USA (USPTO), Japan (JPO), and Europe (EPO) were compared. 

**Figure 6:**
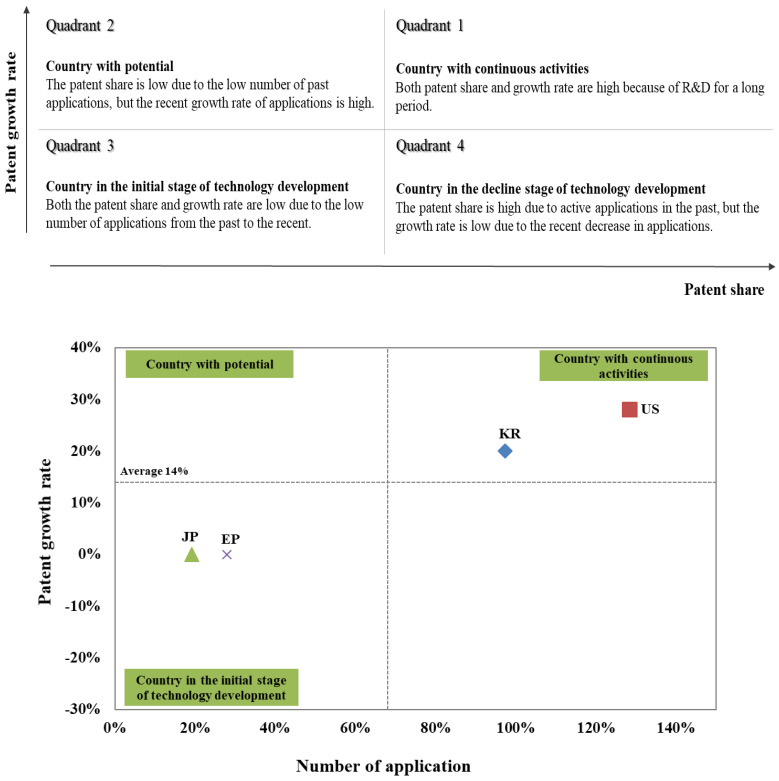
Portfolio analysis interpretation according to patent share and growth rate (by country).

The characteristics of the relative application trend in each major nation can be found by concurrently analyzing the recent patent document growth rate and application share compared to the past for each major country (Figure 6). The patent share is the ratio of the number of patents for each given technology to the total number of patents. The patent growth rate is the 10-year average value of the compound annual growth rate (CAGR) of patents. The patent document trend and technical characteristics based on the location of the specific technology were assessed by presenting each value of the patent share and the growth rate by major countries on the *X*-axis and *Y*-axis. If the country’s patent share and growth rate are in the first quadrant of the graph, it is assumed that it has constantly active patent documents. If it is in the second quadrant, it is assumed that patent documents have become active recently. If it is in the third quadrant, the country’s technology is considered to be in its early stages. If it is in the fourth quadrant, it is assumed that patent documents are recently declining. However, the analysis based on each major country’s patent share and growth rate is a relative comparison and should not be interpreted as an absolute interpretation of the target technology.

South Korea (KIPO) and the USA (USPTO) are considered to have the potential for the development of the target technologies compared to Japan (JPO) and Europe (EPO) due to a rapid increase in patent documents and an increase in patent share in the recent period. In contrast, Japan (JPO) and Europe (EPO) are considered to be in the initial stage of technology development. 

## 4. Conclusions

Research showed objective information by reviewing patent document status, identifying R&D trends and key applicants by nation, and understanding current patent technology trends. The patents were analyzed with a focus on technologies that provide solutions to technological issues by borrowing design features from nature or the features of living organisms. The technical scope of this report included the field of technologies and materials, a domain of ecological imitation technologies that mimic conditions similar to those found in ecology. Furthermore, the technological scope of this report included robot machines and gadgets modeled after certain creatures and ecological elements. 

When the entire patent trend in target technologies is examined, a dramatic growth trend can be observed, beginning in the year 2000. In the patent trends relating to target technologies, some areas constantly grow. The quick growth of patent documents in South Korea and the United States has led the overall trend since the mid-2000s, showing a rising tendency from the mid-2000s. In Japan and Europe, however, the number of applications is undergoing constant growing and declining cycles. However, it does not appear to have a substantial impact on the overall trend.

The number of patents filed for target technologies by country was 163 cases for South Korea (KIPO), 137 cases for the United States (USPTO), 43 cases for Japan (JPO), and 20 cases for Europe (EPO). The number and status of patent documents filed by locals and foreigners vary by country. The ratio and current state of patent documents filed by locals and foreigners in South Korea (KIPO) was 93% (152 cases) and 7% (11 cases), respectively. The growth stage of target technologies based on patented technologies can be understood as an increase in both the number of applications and applicants on the graph. Particularly, the number of applications and applicants increased dramatically from Section 4 to Section 5. Overall, the graph shows the shape of the growth stage.

South Korea (KIPO) and the United States (USPTO) are expected to have the potential for technology development in the target technologies compared to Japan (JPO) and Europe (EPO), owing to the rapid increase in patent documents in the recent period. In contrast, Japan (JPO) and Europe (EPO) are considered to be in the initial stage of technology development. 

When considering the relative technological prospects of major countries, it is evident that Korea and the United States emerge as nations with anticipated sustained technological development activities due to the increasing patent applications. On the other hand, Europe and Japan, relatively, exhibit lower patent growth rates and patent occupancy, indicating their roles as countries for technology adoption rather than extensive technological development. Of course, the patent trend analysis in this study is a relative comparison among major countries, and it cannot be considered an absolute interpretation of the respective technologies.

## Data Availability

The author confirm that the data supporting the findings of this study are available within the article.

## Supplementary Materials

The following supporting information can be downloaded at https://www.mdpi.com/article/10.3390/biomimetics8030288/s1: Table S1: The final search query; Table S2: Representative patents; Table S3: The status of domestic and foreign patent applications in major countries.
