# Supplementary material for: Biomimicry Industry and Patent Trends
Source: Biomimetics (Basel). 2023 Jul 3;8(3):288. doi: 10.3390/biomimetics8030288 (PMC10807642; doi:10.3390/biomimetics8030288)
Supplement: Supplementary file 1 [file biomimetics-08-00288-s001.zip › Supplementary Materials Table S1.pdf]

The final search query

Classification	Search query
1st Search query (base words)	body* bodies* nature* ecolog* ecosystem* life* organism* animal* plant* bio*).ti.) AND biomimetic* bionics* biognosis* biomimicry* bio-mimetic* bio-nics* bio-gnosis* bio-mimicry* near2 imitat* copy* emulat* mimic* impersonat* inspirat* motiv* mimetic*)))).ti,ab,bt.
2nd Search query (keyword)	insect* bug* reptil* amphibian* batrachian* arthropod* crustace* mollus* invertebrate* vertebrate* annelid* echinoderm* flatworms* platyhelminth* coelenterate* seaweed* algae* phaeophyta* chlorophyceae* rhodophyceae* bird* fowl* fish* ichthyolog* rodent* primates* mammal* clam* shellfish* shell-fish* "shell fish*" poultry* chondrichthyes* teleoistei* cephalopod* fruit* vegetable* nut* angiosperm* monocotyledon* monocotyledonous* dicotyledon* dicotyledonous* gymnosperm* phanerogam* broadleaf* needleleaf* spermatophyte* perenn* therophyte* near3 (biomimetic* bionics* biognosis* biomimicry* bio-mimetic* bio-nics* bio-gnosis* bio-mimicry* ((bio*) near2 (imitat* copy* emulat* mimic* impersonat* inspirat* motiv* mimetic*)))).ti,ab,bt.
3rd Search query (keyword expansion)	((beetle* eoleoptera* ladybug* ladybird* coccinellidae* "stag bettel*" lucanus* scarab* chafer* long-horned* longicorn* cerambycidae* buprestid* firefly* "lightning bug*" lampyridae* honeybee* apidae* wasp* hornet* stinkbug* "shield bug*" soldierbug* pentatomid* aphid* "ant" formicidae* butterfly* lepidoptera* "fly" drosophila* diptera* mosquito* mantis* moth* flea* siphonaptera* aphaniptera* walkingstick* "stick insect*" phraortes* cockroach* roach* "giant water bug*" lethocerus* "water strider*" "pond skater*" gerridae* grasshopper* locust* orthopteron* dragonfly* odonata* earwig* dermaptera* cricket* gryllidae* clear-tonned* cicada* hemiptera* cimex* tettigonidae* katydid* horsefly* gadfly* tabanidae* mayfly* ephemeroptera* "mole cricket*" gryllotalpidae* "camel cricket*" rhaphidophoridae* snake* pholidota* lizard* lacertilian* turtle* tortoise* chelinia* testudines* chameleon* crocod* alligator* iguan* terrapin* soft-shelled* trionychidae* salamander* hynobiidae* frog* salientia* toad* spider* araneae* scorpion* nite* tick* acarina* millipede* myriapod* wireworm* diplopoda* centipede* chilopod* scutigeridae* crab* brachyura* crawfish* crayfish* cambaridae* "hermit crab*" shrimp* prawn* barnacle* balanidae* barnacles* "sow bug*" isopoda* krill* "water flea*" octopus* octopoda* cuttlefish* sepioidae* snail* slug* escargot* bradybaenidae* "freshwater snail*" "mud snail*" "pond snail*" gastropod* conch* "turban shell*" "wreath shell*" "top shell*" squid* teuthoidae* "baby octopus*" loliginidae* mussel* mytiloida* oyster* ostreoida* scallop* scollop* pectinidae* baekhab* veneroida* cockle* corbicula* "pen shell*" "razor shell*" "fan shell*" pinna* "small octopus*" limpet* patellogastropoda* abalone* "ear shell*" earthworm* lumbricus* leech* hirudinidae* lugworm* polychaeta* starfish* asteriidae* "sea urchin*" "sea chestnut*" echinoida* "sea cucumber*" holothuroidea* "sea squirt*" pyuridae* "warty sea squirt*" styelidae* "sea anemone*" metridium* coral* jellysifh* "sea jelly*" scyphozoa* kelp* lamionariales* "sea mustard*" alariaceae* fusiformis* gulfweed* fucales* "green laver*" "green algae*" "seaweed fulvescens*" laver* "agar-agar*" "cylon moss*" gelidiales* eel* shark* megachasmidae* ray* stingray* rajidae* rajiformes* catfish* siluriformes* siluridae* "gray mullet*" mugiliformes* mugilidae* mullets* snakehead* "snakeheaded fish*" channidae* "japanese spanish mackerel*" cavalla* smelt* "pond smelt*" sparling* gopher* rockfish* blowfish* fugu* tetraodontiformes* puffer* anglerfish* monkfish* lophiiformes* lophiidae* goosefish* tuna* "clark's anemonesifh*" anemonefish* clownfish* snapper* "sea bream*" "gold bream*" sparidae* porgy* hairfish* cutlassfish* trichiuridae* carp* anabantid* filefish* leatherfish* halibut* plaice* flatfish* sole* pleuronectiformes* pleuronectidae* "righteye flounders*" cypriniformes* cyprinidae* gobiidae* luciogobius* gymnogobius* gobies* "flying fish*" exocoetidae* "scorpion fish*" scorpaeniformes* herring* clupeniformes* clupeidae* "clupeoid fish*" "pudding wife*" wrasses* "horse mackerel*" saurel* carangidae* salmon* salmoniformes* salmonidae* cod* gadiformes* gadidae* pollack* anchovy* engraulidae* mackerel* scombridae* cavally* bass* perch* perciformes* moronidae* croaker* "sciaenoid fish*" sciaenidae* croakers* ricefish* minnow* adrianichthyoidae* spearfish* marlin* istiophoridae* dorado* coryphaena* coryphaenidae* halibut* paralichthyidae* butt* moray* muraenidae* "pacific saury*" "mackerel pike*" scomberesocidae* saury* owl* strigiformes* strigidae* strigiform* dove* pigeon* columbidae* hummingbird* rackettail* saberwing*

The final search query

	thornbill* penguin* sphenisciformes* heron* "japanese crane*" "red-crowned crane*" gruiformes* gruidae* cuckoo* stork* ciconiiforme* ciconiidae* ostrich* struthioniformes* struthionidae* plover* jaana* charadriidae* albatross* diomedeidae* cormorant* snipe* longbill* charadriiformes* scolopacidae* "snow grouse*" ptarmigan* "sacred ibis*" threskiornithidae* flamingo* seagull* gull* laridae* chicken* hen* rooster* cock* leipoa* goose* anseriformes* duck* drake* duckling* hawk* falcon* falconidae* kite* eagle* crake* "water rail*" fucaceae* rallidae* caprimulgiformes* caprimulgidae* "little grebe*" dabchick* podicipediformes* podicipedidae* kiwi* pelican* egret* "white heron*" ardeid* quail* "scops owl*" cuculidae* sparrow* passeriform* passeridae* swallow* hirundinidae* hamster* squirrel* chipmunk* sciurine* beaver* nutria* mouse* rat* rodentia* muridae* rabbit* lagomorph* leporidae* "huinea pig*" cavy* porcupine* hedgehog* erinaceomorpha* erinaceidae* skunk* mephitidae* gorilla* orangutan* monkey* bat* bear* ursid* elephant* whale* cetacea* dolphin* delphinoid* deer* cervid* cat* felidae* dog* sloth* wolf* lion* kangaroo* diprotodontia* potoroo* mactopodidae* marsupialia* koala* phascoloarctidae* "roe deer*" reindeer* "water deer*" leopard* puma* tiger* jaguar* cheetah* rhinoceros* rhinocerotidae* cow* bovidae* zebra* zebrule* zonkey* hippo* hippopotamidae* lamb* sheep* camel* camelid boar* suidae* pig* porcine* otter* weasel* mustelidae* musteline* "sea otter*" "sea lion*" otariidae* "elephant seal*" "sea elephant*" walrus* "odobenus rosmarus*" "true seal*" "largha seal*" "phoca largha*" phoca* phocidae* seal* horse* equidae* chlorine* goat* pup* hyena* hyaenadae* fox* coyote* "sea pig*" dugong* dugongidae* badger* meerkat* mierkat* armadillo* dasypodidae* mole* mogera* talpidea* donkey* mule* lama* buffalo* bubaline* jujube* zizyphus* plum* peach* apricot* prunus* cherry* prune* strawberry* fragaria* rubus* mango* fig* syconus* banana* pomegranate* punicaceae* pineapple* bromeliaceae* grape* vitis* viraceae* persimmon* tangerine* mandarin* orange* "citrus fruit*" rutaceae* pear* apple* pomaceous* "grape fruit*" grapefruit* avocado* tomato* lycopersicon* "oriental melon*" cucurbitaceae* watermelon* melon* peanut* chestnut* almond* "pine nut*" pistachio* walnut* "bog rhubarb*" butterbur* coltsfoot* dropwort* apiales* lovage* "chinese cabbage*" "napa cabbage*" lettuce* salary* spinach* "crown daisy*" yellowweed* cabbage* aralia* spikenard* "garlic scape*" leek* alliaceae* "bamboo shoot*" "green onion*" carrot* radish* ginger* zingiberales* zingiberaceous* zingiberaceae* "lotus root*" burdock* taro* aroideous* garlic* onion* eggplant* aubergine* bightshade* solanales* solanaceae* pepper* capsicum* gourd* cucubitales* cucurbitaceae* cucumber* cucumis* paprika* pumpkin* broccoli* ginseng*) near3 (biomimetic* bionics* biognosis* biomimicry* bio-mimetic* bio-nics* bio-gnosis* bio-mimicry* ((bio*) near2 (imitat* copy* emulat* mimic* impersonat* inspirat* motiv* mimetic*)))).ti,ab,bt.
4th Search query (broadening the scope of the species list)	((“about 50,000 keywords terms in National list of species of Korea”) near3 (biomimetic* bionics* biognosis* biomimicry* bio-mimetic* bio-nics* bio-gnosis* bio-mimicry* ((bio*) near2 (imitat* copy* emulat* mimic* impersonat* inspirat* motiv* mimetic*)))).ti,ab,bt.

*Source of 50,000 keywords: National list of species of Korea. Online at http://kbr.go.kr/accessed on (data of access)
