# Supplementary material for: Biomimicry Industry and Patent Trends
Source: Biomimetics (Basel). 2023 Jul 3;8(3):288. doi: 10.3390/biomimetics8030288 (PMC10807642; doi:10.3390/biomimetics8030288)
Supplement: Supplementary file 1 [file biomimetics-08-00288-s001.zip › Supplementary Materials Table S2.pdf]

Country code	Patent kind code	Application number	Application date	Registration number	Registration date
KR	A	10-2013-0157260	2013-12-17		
KR	B1	10-2001-0071488	2001-11-16	10-0451984	2004-09-30
KR	A	10-2002-0018111	2002-03-30		
KR	B1	10-2002-0055578	2002-09-13	10-0476462	2005-03-04
KR	B1	10-2003-0087242	2003-12-03	10-0540180	2005-12-23
KR	A	10-2004-0000211	2004-01-05		
KR	B1	10-2004-0020122	2004-03-24	10-0515031	2005-09-07
KR	A	10-2006-7016269	2005-01-12		
KR	B1	10-2005-0022169	2005-03-17	10-0678987	2007-01-30
KR	B1	10-2005-0133749	2005-12-29	10-0746878	2007-08-01
KR	B1	10-2007-0028280	2007-03-22	10-1237960	2013-02-21
KR	A	10-2007-0088590	2007-08-31		
KR	B1	10-2007-0088588	2007-08-31	10-0881287	2009-01-23
KR	B1	10-2007-0088589	2007-08-31	10-0881288	2009-01-23
KR	B1	10-2008-0034300	2008-04-14	10-1049220	2011-07-07
KR	B1	10-2008-0040865	2008-04-30	10-0928330	2009-11-17
KR	B1	10-2008-0044391	2008-05-14	10-1457147	2014-10-27
KR	B1	10-2008-0067082	2008-07-10	10-0951915	2010-04-01
KR	B1	10-2008-0076860	2008-08-06	10-1012998	2011-01-27
KR	B1	10-2008-0095352	2008-09-29	10-1006547	2010-12-30
KR	B1	10-2008-0108929	2008-11-04	10-1073224	2011-10-06
KR	B1	10-2008-0114805	2008-11-18	10-1059481	2011-08-19
KR	B1	10-2008-0120670	2008-12-01	10-0976866	2010-08-12
KR	B1	10-2008-0127249	2008-12-15	10-0970265	2010-07-07
KR	B1	10-2009-0001673	2009-01-08	10-1139354	2012-04-17
KR	A	10-2009-0003559	2009-01-16		
KR	A	10-2010-7019404	2009-01-29		
KR	B1	10-2009-0122838	2009-12-11	10-1200799	2012-11-07
KR	A	10-2009-0011280	2009-02-12		
KR	B1	10-2009-0090575	2009-09-24	10-1197117	2012-10-29
KR	B1	10-2009-0092295	2009-09-29	10-1094443	2011-12-08
KR	A	10-2010-0032050	2010-04-07		
KR	B1	10-2010-0035175	2010-04-16	10-1182287	2012-09-06
KR	B1	10-2010-0057302	2010-06-16	10-1198076	2012-10-31
KR	B1	10-2010-0074714	2010-08-02	10-1221558	2013-01-07
KR	B1	10-2010-0095580	2010-09-30	10-1217765	2012-12-26
KR	B1	10-2010-0107109	2010-10-29	10-1769553	2017-08-11
KR	B1	10-2010-0114566	2010-11-17	10-1761846	2017-07-20
KR	B1	10-2010-0117452	2010-11-24	10-1258755	2013-04-22
KR	A	10-2012-7018324	2010-12-15		
KR	B1	10-2010-0137829	2010-12-29	10-1685646	2016-12-06
KR	B1	10-2011-0020198	2011-03-08	10-1276706	2013-06-13
KR	B1	10-2011-0031026	2011-04-05	10-1377743	2014-03-17
KR	B1	10-2011-0034266	2011-04-13	10-1317426	2013-10-02
KR	B1	10-2011-0034256	2011-04-13	10-1254112	2013-04-08
KR	A	10-2011-0086106	2011-08-27		
KR	B1	10-2011-0098165	2011-09-28	10-1281255	2013-06-26
KR	A	10-2011-0122416	2011-11-22		
KR	B1	10-2011-0127525	2011-12-01	10-1327889	2013-11-05
KR	A	10-2012-0002163	2012-01-06		
KR	B1	10-2012-0026709	2012-03-15	10-1357301	2014-01-21
KR	B1	10-2012-0043299	2012-04-25	10-1374004	2014-03-06
KR	A	10-2012-0050126	2012-05-11		
KR	A	10-2012-0054202	2012-05-22		
KR	B1	10-2012-0055809	2012-05-25	10-1388713	2014-04-17
KR	B1	10-2014-7003115	2012-07-09	10-1576117	2015-12-03
KR	B1	10-2012-0123818	2012-11-02	10-1425510	2014-07-25
KR	B1	10-2012-0126679	2012-11-09	10-1363873	2014-02-11
KR	B1	10-2012-0128817	2012-11-14	10-1361491	2014-02-05
KR	B1	10-2012-0129257	2012-11-15	10-1402735	2014-05-27
KR	B1	10-2012-0129293	2012-11-15	10-1400095	2014-05-21

KR	B1	10-2012-0136314	2012-11-28	10-1448870	2014-10-01
KR	A	10-2013-0007737	2013-01-23		
KR	B1	10-2013-0015693	2013-02-14	10-1510876	2015-04-03
KR	B1	10-2013-0036144	2013-04-03	10-1477687	2014-12-23
KR	B1	10-2013-0066087	2013-06-10	10-1295940	2013-08-06
KR	B1	10-2013-0090368	2013-07-30	10-1488247	2015-01-26
KR	A	10-2001-0010629	2001-02-23		
KR	B1	10-2001-0071488	2001-11-16	10-0451984	2004-09-30
KR	A	10-2002-0018111	2002-03-30		
KR	B1	10-2005-0133749	2005-12-29	10-0746878	2007-08-01
KR	B1	10-2002-0055578	2002-09-13	10-0476462	2005-03-04
KR	B1	10-2003-0087242	2003-12-03	10-0540180	2005-12-23
KR	A	10-2004-0000211	2004-01-05		
KR	B1	10-2004-0020122	2004-03-24	10-0515031	2005-09-07
KR	A	10-2006-7016269	2005-01-12		
KR	B1	10-2005-0022169	2005-03-17	10-0678987	2007-01-30
KR	B1	10-2007-0028280	2007-03-22	10-1237960	2013-02-21
KR	A	10-2007-0088590	2007-08-31		
KR	B1	10-2007-0088588	2007-08-31	10-0881287	2009-01-23
KR	B1	10-2007-0088589	2007-08-31	10-0881288	2009-01-23
KR	B1	10-2008-0034300	2008-04-14	10-1049220	2011-07-07
KR	B1	10-2008-0040865	2008-04-30	10-0928330	2009-11-17
KR	B1	10-2008-0044391	2008-05-14	10-1457147	2014-10-27
KR	B1	10-2008-0067082	2008-07-10	10-0951915	2010-04-01
KR	B1	10-2008-0076860	2008-08-06	10-1012998	2011-01-27
KR	B1	10-2008-0095352	2008-09-29	10-1006547	2010-12-30
KR	B1	10-2008-0108929	2008-11-04	10-1073224	2011-10-06
KR	B1	10-2008-0114805	2008-11-18	10-1059481	2011-08-19
KR	B1	10-2008-0120670	2008-12-01	10-0976866	2010-08-12
KR	B1	10-2008-0127249	2008-12-15	10-0970265	2010-07-07
KR	B1	10-2009-0001673	2009-01-08	10-1139354	2012-04-17
KR	A	10-2009-0003559	2009-01-16		
KR	A	10-2010-7019404	2009-01-29		
KR	A	10-2009-0011280	2009-02-12		
KR	B1	10-2009-0092295	2009-09-29	10-1094443	2011-12-08
KR	B1	10-2009-0090575	2009-09-24	10-1197117	2012-10-29
KR	B1	10-2010-0074714	2010-08-02	10-1221558	2013-01-07
KR	B1	10-2010-0095580	2010-09-30	10-1217765	2012-12-26
KR	B1	10-2010-0107109	2010-10-29	10-1769553	2017-08-11
KR	B1	10-2010-0114566	2010-11-17	10-1761846	2017-07-20
KR	B1	10-2010-0117452	2010-11-24	10-1258755	2013-04-22
KR	A	10-2012-7018324	2010-12-15		
KR	B1	10-2010-0137829	2010-12-29	10-1685646	2016-12-06
KR	B1	10-2011-0020198	2011-03-08	10-1276706	2013-06-13
KR	B1	10-2011-0031026	2011-04-05	10-1377743	2014-03-17
KR	B1	10-2011-0034266	2011-04-13	10-1317426	2013-10-02
KR	B1	10-2011-0034256	2011-04-13	10-1254112	2013-04-08
KR	A	10-2011-0086106	2011-08-27		
KR	B1	10-2011-0098165	2011-09-28	10-1281255	2013-06-26
KR	A	10-2011-0122416	2011-11-22		
KR	B1	10-2011-0127525	2011-12-01	10-1327889	2013-11-05
KR	A	10-2012-0002163	2012-01-06		
KR	B1	10-2009-0122838	2009-12-11	10-1200799	2012-11-07
KR	A	10-2010-0032050	2010-04-07		
KR	B1	10-2010-0035175	2010-04-16	10-1182287	2012-09-06
KR	B1	10-2010-0057302	2010-06-16	10-1198076	2012-10-31
KR	B1	10-2012-0026709	2012-03-15	10-1357301	2014-01-21
KR	B1	10-2012-0043299	2012-04-25	10-1374004	2014-03-06
KR	A	10-2012-0050126	2012-05-11		
KR	A	10-2012-0054202	2012-05-22		
KR	B1	10-2012-0055809	2012-05-25	10-1388713	2014-04-17
KR	B1	10-2014-7003115	2012-07-09	10-1576117	2015-12-03

KR	B1	10-2012-0123818	2012-11-02	10-1425510	2014-07-25
KR	B1	10-2012-0126679	2012-11-09	10-1363873	2014-02-11
KR	B1	10-2012-0128817	2012-11-14	10-1361491	2014-02-05
KR	B1	10-2012-0129257	2012-11-15	10-1402735	2014-05-27
KR	B1	10-2012-0129293	2012-11-15	10-1400095	2014-05-21
KR	B1	10-2012-0136314	2012-11-28	10-1448870	2014-10-01
KR	A	10-2013-0007737	2013-01-23		
KR	B1	10-2013-0015693	2013-02-14	10-1510876	2015-04-03
KR	B1	10-2013-0036144	2013-04-03	10-1477687	2014-12-23
KR	B1	10-2013-0066087	2013-06-10	10-1295940	2013-08-06
KR	B1	10-2013-0090368	2013-07-30	10-1488247	2015-01-26
KR	B1	10-2013-0164154	2013-12-26	10-1453221	2014-10-14
KR	B1	10-2014-0006079	2014-01-17	10-1579547	2015-12-16
KR	B1	10-2014-0040662	2014-04-04	10-1554490	2015-09-15
KR	B1	10-2014-0063456	2014-05-27	10-1487839	2015-01-23
KR	B1	10-2014-0070928	2014-06-11	10-1605247	2016-03-15
KR	B1	10-2014-0102601	2014-08-08	10-1484961	2015-01-15
KR	B1	10-2014-0129884	2014-09-29	10-1608039	2016-03-25
KR	B1	10-2014-0139188	2014-10-15	10-1665191	2016-10-05
KR	B1	10-2014-0164673	2014-11-24	10-1684380	2016-12-02
KR	A	10-2016-7017198	2014-11-24		
KR	B1	10-2015-0023844	2015-02-17	10-1724820	2017-04-03
KR	B1	10-2015-0030853	2015-03-05	10-1638340	2016-07-05
KR	B1	10-2015-0035270	2015-03-13	10-1722503	2017-03-28
KR	B1	10-2015-0042193	2015-03-26	10-1554528	2015-09-15
KR	B1	10-2015-0048846	2015-04-07	10-2246902	2021-04-26
KR	B1	10-2015-0065761	2015-05-12	10-1685916	2016-12-07
KR	A	10-2015-0113799	2015-08-12		
KR	A	10-2015-0114798	2015-08-13		
KR	A	10-2015-0114806	2015-08-13		
KR	B1	10-2015-0129801	2015-09-14	10-1787196	2017-10-11
KR	B1	10-2015-0139026	2015-10-02	10-1744721	2017-06-01
KR	B1	10-2015-0149469	2015-10-27	10-1741802	2017-05-24
KR	A	10-2015-0155857	2015-11-06		
KR	A	10-2015-0174672	2015-12-09		
KR	A	10-2018-7021531	2015-12-30		
KR	B1	10-2016-0001333	2016-01-06	10-1738883	2017-05-17
KR	A	10-2017-7023126	2016-01-20		
KR	B1	10-2016-0013069	2016-02-02	10-1815227	2017-12-28
KR	B1	10-2016-0021105	2016-02-23	10-1838534	2018-03-08
KR	B1	10-2016-0062966	2016-05-23	10-1675335	2016-11-07
KR	B1	10-2016-0051876	2016-04-28	10-1831613	2018-02-19
KR	A	10-2018-7010129	2016-06-30		
KR	B1	10-2016-0106210	2016-08-22	10-1954410	2019-02-26
KR	B1	10-2016-0130446	2016-10-10	10-1840621	2018-03-15
KR	B1	10-2016-0170094	2016-12-14	10-1886930	2018-08-02
KR	A	10-2017-0002398	2017-01-06		
KR	B1	10-2017-0006404	2017-01-13	10-1767867	2017-08-07
KR	B1	10-2017-0014548	2017-02-01	10-1936492	2019-01-02
KR	B1	10-2017-0021111	2017-02-16	10-1919906	2018-11-13
KR	A	10-2017-0022044	2017-02-20		
KR	B1	10-2017-0027769	2017-03-03	10-1889484	2018-08-10
KR	B1	10-2017-0046160	2017-04-10	10-1845822	2018-03-30
KR	B1	10-2017-0074794	2017-06-14	10-1904663	2018-09-27
KR	B1	10-2017-0074795	2017-06-14	10-1904664	2018-09-27
KR	B1	10-2017-0086046	2017-07-06	10-1881288	2018-07-18
KR	B1	10-2017-0088660	2017-07-12	10-1820632	2018-01-15
KR	B1	10-2017-0090532	2017-07-17	10-2097449	2020-03-31
KR	A	10-2017-0144933	2017-11-01		
KR	B1	10-2017-0148941	2017-11-09	10-2064915	2020-01-06
KR	B1	10-2017-0152185	2017-11-15	10-2054339	2019-12-04
KR	B1	10-2018-0011453	2018-01-30	10-2023035	2019-09-11

KR	B1	10-2018-0014250	2018-02-05	10-2015040	2019-08-21
KR	B1	10-2018-0017847	2018-02-13	10-2073828	2020-01-30
KR	B1	10-2018-0034358	2018-03-26	10-2054580	2019-12-04
KR	A	10-2019-7035639	2018-05-02		
KR	B1	10-2018-0057997	2018-05-21	10-1882652	2018-07-20
KR	B1	10-2018-0073279	2018-06-26	10-1958759	2019-03-11
KR	B1	10-2018-0075172	2018-06-29	10-2182310	2020-11-18
KR	B1	10-2018-0085197	2018-07-23	10-2071648	2020-01-22
KR	B1	10-2018-0086354	2018-07-25	10-2271744	2021-06-25
KR	B1	10-2018-0086997	2018-07-26	10-1931917	2018-12-17
KR	B1	10-2018-0089350	2018-07-31	10-2076619	2020-02-06
KR	A	10-2021-7007258	2018-08-27		
KR	B1	10-2018-0125914	2018-10-22	10-2080609	2020-02-18
KR	A	10-2018-0137910	2018-11-12		
KR	B1	10-2018-0148384	2018-11-27	10-2174498	2020-10-29
KR	B1	10-2018-0165234	2018-12-19	10-2154907	2020-09-04
KR	A	10-2018-0169172	2018-12-26		
KR	B1	10-2018-0173654	2018-12-31	10-2289463	2021-08-06
KR	B1	10-2019-0005285	2019-01-15	10-2186049	2020-11-27
KR	B1	10-2019-0005286	2019-01-15	10-2182067	2020-11-17
KR	A	10-2019-0024957	2019-03-04		
KR	B1	10-2019-0033858	2019-03-25	10-2214475	2021-02-03
KR	B1	10-2019-0042705	2019-04-11	10-2134474	2020-07-08
KR	B1	10-2019-0068498	2019-06-11	10-2238394	2021-04-05
KR	B1	10-2019-0072164	2019-06-18	10-2177754	2020-11-05
KR	B1	10-2019-0082123	2019-07-08	10-2228882	2021-03-11
KR	B1	10-2019-0089337	2019-07-24	10-2226435	2021-03-05
KR	B1	10-2019-0104294	2019-08-26	10-2315515	2021-10-15
KR	B1	10-2019-0108851	2019-09-03	10-2272831	2021-06-29
KR	A	10-2019-0140511	2019-11-05		
KR	B1	10-2019-0162713	2019-12-09	10-2231057	2021-03-17
KR	A	10-2020-0015182	2020-02-07		
KR	B1	10-2019-0177957	2019-12-30	10-2333892	2021-11-29
KR	B1	10-2020-0036682	2020-03-26	10-2330922	2021-11-22
KR	B1	10-2020-0040662	2020-04-03	10-2310625	2021-10-01
KR	B1	10-2020-0045413	2020-04-14	10-2276602	2021-07-07
KR	A	10-2020-0046299	2020-04-16		
KR	B1	10-2020-0051808	2020-04-28	10-2333694	2021-11-26
KR	B1	10-2020-0056513	2020-05-12	10-2261754	2021-06-01
KR	B1	10-2020-0057647	2020-05-14	10-2322217	2021-11-01
KR	A	10-2020-0059730	2020-05-19		
KR	B1	10-2020-0088666	2020-07-17	10-2249949	2021-05-03
KR	A	10-2021-0059847	2021-05-10		
KR	B1	10-2021-0066784	2021-05-25	10-2335485	2021-12-01
US	B2	13/632604	2012-10-01	9463393	2016-10-11
US	B2	13/720773	2012-12-19	8960595	2015-02-24
US	B2	14/383038	2013-03-07	9938368	2018-04-10
US	A1	13/917615	2013-06-13		
US	A	06/474996	1983-03-14	4527000	1985-07-02
US	A	06/566516	1983-12-29	4544504	1985-10-01
US	A	06/593058	1984-03-26	4548804	1985-10-22
US	A	06/704442	1985-02-22	4722839	1988-02-02
US	A	06/719881	1985-04-04	4861714	1989-08-29
US	A	06/899301	1986-08-21	4752051	1988-06-21
US	A	06/932088	1986-11-18	4863856	1989-09-05
US	A	07/043956	1987-04-29	4755005	1988-07-05
US	A	07/333831	1989-04-05	5100783	1992-03-31
US	A	07/393656	1989-08-14	4997753	1991-03-05
US	A	07/787579	1991-11-04	5465523	1995-11-14
US	A	07/893232	1992-06-03	5519165	1996-05-21
US	A	08/190390	1994-02-02	5497671	1996-03-12
US	A	08/371289	1995-01-11	5495690	1996-03-05

US	A	08/563122	1995-11-29	5659994	1997-08-26
US	A	08/562705	1996-04-10	5678730	1997-10-21
US	A	08/856522	1997-05-14	5993406	1999-11-30
US	A	08/889340	1997-07-08	5941007	1999-08-24
US	A	08/893964	1997-07-16	5906525	1999-05-25
US	A	08/932573	1997-09-19	5879387	1999-03-09
US	A	08/964876	1997-11-05	6004444	1999-12-21
US	A	09/076479	1998-05-12	6038804	2000-03-21
US	B1	09/089602	1998-06-02	6260795	2001-07-17
US	B1	09/291549	1999-04-14	6228248	2001-05-08
US	B1	09/294737	1999-04-19	6379619	2002-04-30
US	A	09/351361	1999-07-12	6053794	2000-04-25
US	B1	09/435984	1999-11-08	6250330	2001-06-26
US	B1	09/456275	1999-12-07	6331178	2001-12-18
US	B1	09/627808	2000-07-28	6537575	2003-03-25
US	B1	09/686608	2000-10-11	6564720	2003-05-20
US	B2	09/846598	2001-04-30	6406101	2002-06-18
US	B2	09/850364	2001-05-07	6547940	2003-04-15
US	A1	09/877903	2001-06-08		
US	B1	09/891083	2001-06-25	6486334	2002-11-26
US	A1	09/893720	2001-06-28		
US	B1	10/060142	2002-02-01	6893307	2005-05-17
US	B2	10/358038	2003-02-03	8048155	2011-11-01
US	B2	10/384039	2003-03-07	6799519	2004-10-05
US	B2	10/411641	2003-04-11	7179356	2007-02-20
US	B2	10/618949	2003-07-14	6915859	2005-07-12
US	B2	10/627967	2003-07-28	7208089	2007-04-24
US	A1	10/631982	2003-07-31		
US	B1	10/677206	2003-10-02	7137465	2006-11-21
US	B1	10/781331	2004-02-17	7083492	2006-08-01
US	B1	10/781172	2004-02-17	7070473	2006-07-04
US	B2	10/810518	2004-03-26	7597885	2009-10-06
US	B1	10/845910	2004-05-14	6997765	2006-02-14
US	B2	10/898673	2004-07-23	7769487	2010-08-03
US	B2	10/928073	2004-08-26	7007609	2006-03-07
US	B1	10/936928	2004-09-09	7039292	2006-05-02
US	A1	10/936066	2004-09-09		
US	A1	11/064607	2005-02-24		
US	B2	11/070723	2005-03-01	7314735	2008-01-01
US	B1	11/085955	2005-03-22	7637795	2009-12-29
US	A1	11/101002	2005-04-07		
US	B2	11/596148	2005-05-10	7540570	2009-06-02
US	B1	11/234095	2005-09-26	7322226	2008-01-29
US	B2	11/816684	2006-02-10	7857831	2010-12-28
US	B1	11/369902	2006-03-07	7302892	2007-12-04
US	A1	11/719880	2005-12-07		
US	B2	11/422388	2006-06-06	8162859	2012-04-24
US	A1	11/559516	2006-11-14		
US	B2	11/732867	2007-04-04	7762362	2010-07-27
US	A1	11/827827	2007-07-13		
US	A1	12/139828	2008-06-16		
US	A1	12/400043	2009-03-09		
US	A1	12/385654	2009-04-15		
US	B2	12/997272	2009-06-10	8545746	2013-10-01
US	B2	12/742088	2008-11-05	8449620	2013-05-28
US	B2	12/342583	2008-12-23	8382029	2013-02-26
US	B2	12/981760	2010-12-30	8472762	2013-06-25
US	B2	12/984947	2011-01-05	9042204	2015-05-26
US	A1	13/165157	2011-06-21		
US	A1	13/450370	2012-04-18		
US	B2	13/451271	2012-04-19	8677587	2014-03-25
US	B2	12/820571	2010-06-22	8494676	2013-07-23

US	B2	13/987921	2013-09-17	9045211	2015-06-02
US	A1	14/108041	2013-12-16		
US	B2	14/135740	2013-12-20	10329863	2019-06-25
US	B1	13/385044	2012-01-28	8286973	2012-10-16
US	A1	13/817253	2012-02-10		
US	B2	14/000191	2012-02-17	9290268	2016-03-22
US	B2	12/342583	2008-12-23	8382029	2013-02-26
US	B1	13/385044	2012-01-28	8286973	2012-10-16
US	A1	13/817253	2012-02-10		
US	B2	14/000191	2012-02-17	9290268	2016-03-22
US	B2	13/632604	2012-10-01	9463393	2016-10-11
US	B2	13/720773	2012-12-19	8960595	2015-02-24
US	B2	14/383038	2013-03-07	9938368	2018-04-10
US	A1	13/917615	2013-06-13		
US	B2	14/025728	2013-09-12	9035558	2015-05-19
US	B2	14/759309	2014-02-19	9745057	2017-08-29
US	B2	14/759321	2014-02-19	9745058	2017-08-29
US	B1	14/268837	2014-05-02	9345240	2016-05-24
US	B2	14/273527	2014-05-08	9763502	2017-09-19
US	B1	14/320931	2014-07-01	9248892	2016-02-02
US	B2	14/525010	2014-10-27	10526086	2020-01-07
US	B2	14/729594	2015-06-03	10212926	2019-02-26
US	B2	15/036813	2015-08-06	9938007	2018-04-10
US	A1	14/836715	2015-08-26		
US	B2	14/866220	2015-09-25	9756846	2017-09-12
US	B1	14/956453	2015-12-02	9616979	2017-04-11
US	B2	15/543164	2015-12-04	10144893	2018-12-04
US	B2	15/094602	2016-04-08	10960267	2021-03-30
US	B2	15/138592	2016-04-26	9661842	2017-05-30
US	B2	15/149167	2016-05-08	10422359	2019-09-24
US	B2	15/755115	2016-05-27	10980605	2021-04-20
US	B2	15/226563	2016-08-02	9775338	2017-10-03
US	B2	15/761855	2016-09-21	10605962	2020-03-31
US	A1	15/272030	2016-09-21		
US	B2	15/291870	2016-10-12	10308375	2019-06-04
US	B2	15/291874	2016-10-12	10220963	2019-03-05
US	B2	15/598630	2017-05-18	10477918	2019-11-19
US	B2	15/610775	2017-06-01	10549361	2020-02-04
US	B1	15/673335	2017-08-09	10212503	2019-02-19
US	A1	15/678262	2017-08-16		
US	B1	15/866349	2018-01-09	10817052	2020-10-27
US	B2	15/888004	2018-02-03	10920800	2021-02-16
US	B2	15/942730	2018-04-02	10610370	2020-04-07
US	B2	15/964752	2018-04-27	11136117	2021-10-05
US	B2	15/969176	2018-05-02	11039823	2021-06-22
US	A1	16/627929	2018-06-25		
US	B2	15/682033	2017-08-21	10195048	2019-02-05
US	B2	16/190118	2018-11-13	11122820	2021-09-21
US	A1	16/240234	2019-01-04		
US	B2	16/241398	2019-01-07	10924837	2021-02-16
US	A1	16/257679	2019-01-25		
US	A1	16/266788	2019-02-04		
US	B2	16/362089	2019-03-22	10548311	2020-02-04
US	B1	16/424762	2019-05-29	10569182	2020-02-25
US	A1	16/432190	2019-06-05		
US	A1	16/452183	2019-06-25		
US	B2	16/531095	2019-08-04	10805732	2020-10-13
US	B1	16/726071	2019-12-23	11142878	2021-10-12
US	A1	16/734341	2020-01-05		
US	A1	16/780896	2020-02-03		
US	B2	16/822381	2020-03-18	11155326	2021-10-26
US	B1	17/069868	2020-10-14	10935986	2021-03-02

US	A1	17/100666	2020-11-20		
US	A1	17/130152	2020-12-22		
US	A1	17/175642	2021-02-13		
JP	A	2002-134252	2002-05-09		
JP	A	2002-133850	2002-05-09		
JP	A	2007-552440	2005-07-07		
JP	A	2005-350639	2005-12-05		
JP	A	2003-559369	2002-12-20		
JP	A	2008-509279	2006-05-02		
JP	B2	2009-507796	2007-04-25	5538881	2014-05-09
JP	A	2007-145415	2007-05-31		
JP	A	2007-145424	2007-05-31		
JP	A	2007-145430	2007-05-31		
JP	B2	2008-250098	2008-09-29	5120645	2012-11-02
JP	B2	2008-254320	2008-09-30	5088580	2012-09-21
JP	A	2009-026022	2009-02-06		
JP	A	2009-194436	2009-08-25		
JP	B2	2011-525252	2009-08-28	5607629	2014-09-05
JP	A	2012-516515	2010-06-23		
JP	A	2012-094986	2012-04-18		
JP	B2	2013-197971	2013-09-25	6171788	2017-07-14
JP	B2	2013-204011	2013-09-30	6171801	2017-07-14
JP	B2	1988-058127	1988-03-11	2791031	1998-06-12
JP	B2	1994-143713	1994-06-27	3243937	2001-10-26
JP	B2	1994-202330	1994-08-26	3712276	2005-08-26
JP	A	1995-087480	1995-03-20		
JP	A	1997-028126	1997-02-12		
JP	A	1998-087334	1998-03-31		
JP	A	1999-098973	1999-04-06		
JP	A	2000-620999	2000-05-26		
JP	A	2000-224117	2000-07-25		
JP	A	2001-184354	2001-06-19		
JP	A	2001-184356	2001-06-19		
JP	A	2002-134252	2002-05-09		
JP	A	2002-133850	2002-05-09		
JP	A	2003-559369	2002-12-20		
JP	A	2007-552440	2005-07-07		
JP	A	2005-350639	2005-12-05		
JP	A	2008-509279	2006-05-02		
JP	B2	2009-507796	2007-04-25	5538881	2014-05-09
JP	A	2007-145415	2007-05-31		
JP	A	2007-145424	2007-05-31		
JP	A	2007-145430	2007-05-31		
JP	B2	2008-250098	2008-09-29	5120645	2012-11-02
JP	B2	2008-254320	2008-09-30	5088580	2012-09-21
JP	A	2009-026022	2009-02-06		
JP	A	2009-194436	2009-08-25		
JP	B2	2011-525252	2009-08-28	5607629	2014-09-05
JP	A	2012-094986	2012-04-18		
JP	A	2012-516515	2010-06-23		
JP	B2	2013-264675	2013-12-20	5694497	2015-02-13
JP	A	2014-102256	2014-05-16		
JP	B2	2015-526214	2014-06-05	6052641	2016-12-09
JP	A	2015-162969	2015-08-20		
JP	A	2017-531543	2015-12-09		
JP	A	2017-550079	2015-12-09		
JP	A	2016-102733	2016-05-23		
JP	B2	2016-126496	2016-06-27	6636869	2019-12-27
JP	B2	2018-528929	2016-08-22	6770576	2020-09-29
JP	A	2017-060705	2017-03-27		
JP	A	2017-092111	2017-05-08		
JP	A	2020-535223	2017-12-31		

JP	A	2020-500159	2018-06-25		
EP	B1	2006-123844	2002-06-14	1745763	2016-08-10
EP	B1	2003-783595	2003-11-17	1684578	2013-01-23
EP	B1	2005-826668	2005-12-07	1861178	2015-11-04
EP	B1	2007-756046	2007-04-25	2010104	2018-09-05
EP	B1	2007-120198	2007-11-07	2057971	2010-06-09
EP	A2	2007-853144	2007-11-20		
EP	B1	2007-845710	2007-11-27	2216430	2013-01-09
EP	B1	2008-841931	2008-10-26	2215330	2014-04-16
EP	A1	2009-174760	2009-11-02		
EP	B1	2010-810832	2010-07-29	2468074	2020-04-22
EP	A2	1985-301685	1985-03-12		
EP	B1	1986-902601	1986-03-26	0217917	1993-12-15
EP	B1	1999-912528	1999-03-17	1161219	2009-01-14
EP	B1	2006-123844	2002-06-14	1745763	2016-08-10
EP	B1	2003-783595	2003-11-17	1684578	2013-01-23
EP	B1	2005-826668	2005-12-07	1861178	2015-11-04
EP	B1	2007-756046	2007-04-25	2010104	2018-09-05
EP	B1	2007-120198	2007-11-07	2057971	2010-06-09
EP	A2	2007-853144	2007-11-20		
EP	B1	2007-845710	2007-11-27	2216430	2013-01-09
EP	B1	2008-841931	2008-10-26	2215330	2014-04-16
EP	A1	2009-174760	2009-11-02		
EP	B1	2010-810832	2010-07-29	2468074	2020-04-22
EP	B1	2014-754397	2014-02-19	2923944	2020-10-14
EP	B1	2016-725012	2016-05-11	3304243	2019-04-10
EP	A2	2017-869771	2017-08-21		
EP	A1	2017-818180	2017-12-22		
EP	A1	2018-828449	2018-06-25		
EP	A1	2018-749099	2018-07-24		
EP	A1	2019-803597	2019-05-16		
