# Supplementary material for: Biomimicry Industry and Patent Trends
Source: Biomimetics (Basel). 2023 Jul 3;8(3):288. doi: 10.3390/biomimetics8030288 (PMC10807642; doi:10.3390/biomimetics8030288)
Supplement: Supplementary file 1 [file biomimetics-08-00288-s001.zip › Supplementary Materials Table S3.pdf]

The status of domestic and foreign patent applications in major countries

Country	KIPO		USPTO		JPO		EPO	
**Total**	287		472		88		93	
**Domestic/ Foreigner**	Domestic	254	Domestic	334	Domestic	42	European	32
	Foreigner	33	Foreigner	138	Foreigner	46	non-European	61
**Numbers of applications by country among foreigners**	US	15	CN	29	US	28	US	47
	DE	3	KR	23	KR	4	KR	4
	DK	2	TW	12	CN	3	JP	3
	JP	2	JP	9	SG	3	CN	3
	CN	2	DE	8	DE	2	TW	2
	Other	9	Other	57	Other	6	Other	2
